# Critical circumferential wavelength of elastic buckling of longitudinal compressed thin-walled cylindrical shells

**DOI:** 10.1038/s41598-023-43696-5

**Published:** 2023-10-04

**Authors:** Ming Ji

**Affiliations:** Kamakura, Japan

**Keywords:** Aerospace engineering, Civil engineering, Mechanical engineering

## Abstract

The classical theory of elastic critical buckling stress works well for slender columns and thin flat plates under compression; however, the situation is different for longitudinally compressed thin-walled circular cylindrical shells, and the issue has plagued us despite considerable efforts over the last 100 years. We noticed that all such buckling analyses thus far, both linear and nonlinear, in terms of the main philosophy, inherited and were confined to Euler’s pioneering solution for the slender column model that focuses on the longitudinal buckling deformation mode and should be classified as the ‘longitudinal open-loop’ eigenmode because the deformations of the two longitudinal ends are physically independent of each other. In view of this, for an ideal linear-elastic buckling model of a thin-walled perfectly circular cylindrical shell under uniform longitudinal compression on the foundation of the longitudinal open-loop eigenmode solution, it is also necessary to consider a ‘circumferential closed-loop’ eigenmode simultaneously to physically avoid violating the reality of its ideal periodic deformation on the entire perimeter and to mathematically redefine the biunique and precise relationship for each distinct eigenmode by the critical circumferential wavelength. Originating from such a case study, the mathematical uniqueness issue hidden in the general solution of the Donnell equation is further discussed. The authenticity of the competing eigenmode characterized by the Koiter circle is also discussed. Furthermore, a preliminary attempt was conducted to interpret the discrepancy between theoretical and experimental buckling loads, mainly initiated by the characteristic type of longitudinally generated circumferential local inward displacement in initial geometric imperfections, using the insights herein.

## Introduction

The classical static, linear, small-deflection theory of elastic buckling of thin-walled perfectly circular cylindrical shells that is paved by Euler’s pioneering investigation way for the elastic stability of an initially straight slender column^1,2^, became simple and clear with the clarification of mechanics concept of its physical model; the many ingenious analyses made by independent pioneers, including Rayleigh, Love, Lorenz, Southwell, von Mises, Flügge, Timoshenko, von Kármán, Donnell, Batdorf, and others, for a full count see Timoshenko and Gere^3^. The mechanics framework with respect to the physical model for the static linear-elastic analysis of thin-walled perfectly circular cylindrical shells is based upon some idealized rational assumptions essentialized by: (a) the thickness of the shell is small compared with the radius of curvature of its mid-surface; (b) the straight lines in the cylinder wall, perpendicular to the undeformed mid-surface, remain straight and perpendicular to the deformed mid-surface and suffer no extension (Love–Kirchhoff hypothesis); (c) strains and displacements so that the quantities of second and higher order terms are neglected in comparison with the first order terms^4^; (d) the stress components normal to mid-surface are small compared with other stress components and they can be neglected in the stretching and bending stress–strain relations of the homogeneous, isotropic linear-elastic plane-stress state based on generalized Hooke’s law^3^; and (e) the displacements are so small that the static equilibrium conditions for the deformed infinitesimal element of the cylindrical shell are the same as if the element were not deformed and the specification of Love’s so-called ‘first approximation’ of the mechanical properties can be applied to the element as if the element were flat^5–7^.

Whether applying the energy method or differential equation method for the elastic buckling analysis of thin-walled perfectly circular cylindrical shells under uniform longitudinal compression, both classical approaches generate an identical eigenvalue problem to find the lowest load for the loss of stability, termed elastic critical buckling stress (ECBS). Simultaneously, as the turning point load of the bifurcation, buckling deflection will at least begin to appear, and the associated eigenmode is the buckling mode. In the early nineteenth century, the same form of the ECBS prediction formula corresponding to axisymmetric buckling and nonaxisymmetric periodic buckling modes was proposed, but it was soon clear that the observed experimental elastic buckling load of a real shell was significantly lower than the predicted value^3^, and revealed that^8,9^: (a) the experimental elastic buckling loads were often much lower than the predictions of the classical theory; (b) there was a wide scatter in the experimental elastic buckling loads for nominally identical specimens, that is, unpredictability; (c) the failures were often catastrophic, that is, unstable and dynamic. This significant and embarrassing discovery has been actively studied for about a century, and conventional wisdom has been made that the single dominant factor of initial geometric imperfections in spite of boundary conditions, small load eccentricities, etc., should be mainly responsible for the occurrence of lower-than-expected ECBS^10^. Two different theoretical approaches for the highly unstable postbuckling behavior and imperfection-sensitive nature of longitudinally compressed thin-walled cylindrical shells with initial geometrical imperfections emerged by Kármán and Tsien^11–13^ and Koiter^14^, respectively, which can be referred to in a review by Hutchinson and Koiter^15^.

It should be noted that all physical models for linear-elastic buckling and nonlinear postbuckling solutions of longitudinally compressed thin-walled cylindrical shells with perfect or imperfect geometry have, to a certain extent, been inherited and confined to Euler’s solution concept for the elastic slender column model; that is, these studies always focus on the solutions of the longitudinal buckling deformation mode. It is self-evident that such buckling models and solutions should be classified as the so-called longitudinal open-loop eigenmode generated on a straight or slightly flexural line because the deformations of the two longitudinal ends are physically independent of each other, similar to the noteworthy eigenmode on the compressed slender column. However, it is also necessary to consider the linear-elastic solution of the nonaxisymmetric periodic buckling deformation mode generated on the circumference. In other words, for a thin-walled perfectly circular cylindrical shell subjected to uniform longitudinal compression or other types of loads in the elastic state, on the foundation of the longitudinal open-loop eigenmode solution, a circumferential closed-loop eigenmode should be added simultaneously to satisfy the idealized continuous circumferential buckling deformation condition, which has a complete integer periodic waveform on its entire perimeter, so as not to violate the reality of circumferential periodicity.

Consequently, herein, still based on the classical static, linear, small-deflection theory of thin-walled perfectly circular cylindrical shells under uniform longitudinal compression, the elastic nonaxisymmetric periodic buckling solution of the circumferential closed-loop eigenmode generated with complete integer periodic waveform on its whole perimeter is presented by utilizing the Donnell equation, in which the geometric concepts of the critical circumferential wavelength or integer wavenumber on whole perimeter and aspect ratio of wave pattern are included and clarified and the biunique and precise elastic critical buckling relationship for each distinct eigenmode is mathematically redefined. Furthermore, based on such a case study, the mathematical uniqueness issue hidden in the general solution of the Donnell equation is discussed, and the rational argument that adding only a circumferential closed-loop model can uniquely satisfy the physical features of elastic nonaxisymmetric periodic buckling of thin-walled perfectly circular cylindrical shells under uniform longitudinal compression is clarified. The authenticity of the competing eigenmode characterized by the Koiter circle is also discussed. Furthermore, in contrast to the previous conventional understanding, a preliminary attempt is made to interpret the discrepancy between theoretical and experimental buckling loads that is mainly initiated by the characteristic type of longitudinally generated circumferential local inward displacement of initial geometric imperfections using the insights herein.

## Fundamental theory

For analytic convenience, with the aid of Donnell’s single simplified equation of eighth-order partial differential expression governing the elastic critical buckling state of a thin-walled perfectly circular cylindrical shell model under uniform longitudinal compression, as well as its definitions of coordinate and displacement component^16^, and the idealized rational assumptions mentioned in the introduction, Donnell’s general equation can be expressed as follows:

1 $$D\cdot {\nabla }^{8}w+\frac{Et}{{r}^{2}}\frac{{\partial }^{4}w}{\partial {x}^{4}}+t\cdot {\sigma }_{x}^{s}\cdot {\nabla }^{4}\left(\frac{{\partial }^{2}w}{\partial {x}^{2}}\right)=0 ,\ \ \ D=\frac{E{t}^{3}}{12\left(1-{\nu }^{2}\right)} ,\ \ \ {\nabla }^{4}={\left({\nabla }^{2}\right)}^{2}={\left(\frac{{\partial }^{2}}{\partial {x}^{2}}+\frac{{\partial }^{2}}{\partial {y}^{2}}\right)}^{2}$$

where ${\nabla }^{2}$ is the Laplacian, and *D* is the flexural stiffness per unit length. The cylindrical shell model of length *L*, radius *r*, and uniform thickness *t* is made of a homogeneous, isotropic, linear-elastic material with Young’s modulus *E* and Poisson’s ratio *ν*, and is only subjected to a uniform longitudinal compressive load *σ*^*s*^_*x*_ (stretching type), with mid-surface displacements *u* in the longitudinal direction (*x*-direction), *v* in the circumferential direction (*y*-direction), and *w* in the radial direction (*r*-direction).

### Longitudinal open-loop eigenmode buckling solutions for thin-walled perfectly circular cylindrical shells under uniform longitudinal compression

Currently, the known classical elastic critical buckling solutions of infinitely long or relatively long, not too short but finite length, thin-walled perfectly circular cylindrical shells under uniform longitudinal compression include axisymmetric form and nonaxisymmetric periodic form. Both such buckling solutions can be classified as longitudinal open-loop eigenmodes because the deformations of the two longitudinal ends are physically independent of each other. The deflection solution *w*(*x*, *y*) of the longitudinal open-loop eigenmode of axisymmetric buckling, which satisfies the boundary conditions of simply support, is a single sinusoidal eigenfunction only relevant to the longitudinal *x*-direction^3^, as follows:

2 $$w={w}_{0}\mathrm{sin}\left(\frac{m\pi x}{L}\right)$$

where *m* denotes the longitudinal buckling wavenumber; *w*_0_ is the radial deflection coefficient of the point on the mid-surface; and the outward direction is positive.

However, the deflection solution *w*(*x*, *y*) of the longitudinal open-loop eigenmode of nonaxisymmetric periodic buckling, which also satisfies the boundary conditions of simply support, is a doubly sinusoidal eigenfunction either along the longitudinal *x*-direction or around the circumferential *y*-direction^3,17^, as follows:

3 $$w={w}_{0}\mathrm{sin}\left(\frac{m\pi x}{L}\right)\mathrm{sin}\left(\frac{\pi y}{{b}^{c}}\right)$$

where *b*^*c*^ = *πr*/*n* is the half-wavelength on the circumference and *n* denotes the circumferential periodic buckling wavenumber (*n* ≥ 4).

Upon substituting Eqs. (2) and (3) into Eq. (1), the axisymmetric ECBS and nonaxisymmetric periodic ECBS with the same form of *σ*^*so*^_*xcri*_ can be derived separately^3,17,18^, as follows:

4 $${\sigma }_{xcri}^{so}=\frac{E}{\sqrt{3\left(1-{\nu }^{2}\right)}}\cdot \frac{t}{r}$$

Equation (4) implies that the ECBS is independent of the number and shape of buckling waves in both the longitudinal and circumferential directions. Nevertheless, in the National Aeronautics and Space Administration (NASA) Space Vehicle Design Criteria (SP-8007)^19^ and European Cooperation for Space Standardization (ECSS) Buckling Structures Handbook^20^, Eq. (4) still plays a critical role in the preliminary analysis and design of thin-walled shells after considering the buckling knockdown factors (KDFs), which were determined by establishing a lower bound to the available experimental data.

The nonaxisymmetric periodic buckling solution of the circumferential closed-loop eigenmode is discussed below, and is closely related to the number and shape of the circumferential periodic buckling wave. This implies that only the axisymmetric ECBS is theoretically true and invariant for both the longitudinal open-loop and circumferential closed-loop eigenmodes.

### Circumferential closed-loop eigenmode buckling solution of a thin-walled perfectly circular cylindrical shell under uniform longitudinal compression

For the nonaxisymmetric deformation with an ideal whole perimeter complete integer periodic waveform, the deflection solution *w*(*x*, *y*) of the circumferential closed-loop eigenmode of nonaxisymmetric periodic buckling, which satisfies the boundary conditions of simply support, is also proposed as a doubly sinusoidal eigenfunction either along the longitudinal *x*-direction or around the circumferential *y*-direction as follows:

5 $$w={w}_{0}\mathrm{sin}\left(\frac{\pi x}{\lambda }\right)\mathrm{sin}\left(\frac{\pi y}{{b}_{n}^{c}}\right)$$

where *λ* = *πr*/*m* is the half-wavelength on the longitude and *b*^*c*^_*n*_ = *πr*/*n* is the half-wavelength on the circumference.

Upon substituting Eq. (5) into Eq. (1) yields the following equation:

6 $${w}_{0}\left\{D{\left[{\left(\frac{\pi }{\lambda }\right)}^{2}+{\left(\frac{\pi }{{b}_{n}^{c}}\right)}^{2}\right]}^{4}+\frac{Et}{{r}^{2}}{\left(\frac{\pi }{\lambda }\right)}^{4}-t\cdot {\sigma }_{x}^{sc}\cdot {\left[{\left(\frac{\pi }{\lambda }\right)}^{2}+{\left(\frac{\pi }{{b}_{n}^{c}}\right)}^{2}\right]}^{2}\cdot {\left(\frac{\pi }{\lambda }\right)}^{2}\right\}\mathrm{sin}\left(\frac{\pi x}{\lambda }\right)\mathrm{sin}\left(\frac{\pi y}{{b}_{n}^{c}}\right)=0$$

The expressions within the braces in Eq. (6) must be zero; thus, the nonaxisymmetric periodic ECBS (*σ*^*sc*^_*xcri*_) is

7 $${\sigma }_{xcri}^{sc}=\frac{{\pi }^{2}E}{12\left(1-{\nu }^{2}\right)}{\left(\frac{t}{{b}_{n}^{c}}\right)}^{2}\cdot \frac{{\left[{\left(\frac{{b}_{n}^{c}}{\lambda }\right)}^{2}+1\right]}^{2}}{{\left(\frac{{b}_{n}^{c}}{\lambda }\right)}^{2}}+\frac{E\cdot {\left({b}_{n}^{c}\right)}^{2}}{{{\pi }^{2}r}^{2}}\cdot \frac{{\left(\frac{{b}_{n}^{c}}{\lambda }\right)}^{2}}{{\left[{\left(\frac{{b}_{n}^{c}}{\lambda }\right)}^{2}+1\right]}^{2}}$$

Equation (7) is identical to Eq. (36), which was derived using the energy approach in the study by Kármán and Tsien^12^, where *b*^*c*^_*n*_/*λ* = *m*/*n* = *μ* is the ‘aspect ratio’ of the buckling wave pattern.

Herein, three values of the aspect ratio are used for exemplifying: *μ* = 1.0, *μ* = 0.5, or *μ* = 2.0, thereby

8 $${\left({\sigma }_{xcri}^{sc}\right)}_{\mu =1}=\frac{{\pi }^{2}E}{3\left(1-{\nu }^{2}\right)}{\left(\frac{t}{{b}_{n}^{c}}\right)}^{2}+\frac{E\cdot {\left({b}_{n}^{c}\right)}^{2}}{4\cdot {{\pi }^{2}r}^{2}}$$

9 $${\left({\sigma }_{xcri}^{sc}\right)}_{\mu =0.5}={\left({\sigma }_{xcri}^{sc}\right)}_{\mu =2}=\frac{6.25\cdot {\pi }^{2}E}{12\left(1-{\nu }^{2}\right)}{\left(\frac{t}{{b}_{n}^{c}}\right)}^{2}+\frac{E\cdot {\left({b}_{n}^{c}\right)}^{2}}{6.25\cdot {{\pi }^{2}r}^{2}}$$

Because *b*^*c*^_*n*_ = *πr*/*n*, Eqs. (8) and (9) can also be rewritten as:

10 $$\frac{{\left({\sigma }_{xcri}^{sc}\right)}_{\mu =1}}{E}=\frac{{n}^{2}}{3\left(1-{\nu }^{2}\right)}{\left(\frac{t}{r}\right)}^{2}+\frac{1}{4\cdot {n}^{2}}$$

11 $$\frac{{\left({\sigma }_{xcri}^{sc}\right)}_{\mu =0.5, \mu =2}}{E}=\frac{{6.25\cdot n}^{2}}{12\left(1-{\nu }^{2}\right)}{\left(\frac{t}{r}\right)}^{2}+\frac{1}{6.25\cdot {n}^{2}}$$

Through Eqs. (4) and (10), we have

12 $$\frac{{\left({\sigma }_{xcri}^{sc}\right)}_{\mu =1}}{E}=\frac{{n}^{2}}{3\left(1-{\nu }^{2}\right)}\cdot {\left(\frac{t}{r}\right)}^{2}+\frac{1}{4\cdot {n}^{2}}=\frac{1}{\sqrt{3\left(1-{\nu }^{2}\right)}}\cdot \frac{t}{r}=\frac{{\sigma }_{xcri}^{so}}{E}$$

By introducing a dimensionless parameter *χ*:

13 $$\chi =\frac{{n}^{2}}{\sqrt{3\left(1-{\nu }^{2}\right)}}\cdot \frac{t}{r}$$

Solve Eq. (12) to obtain

14 $$4{\chi }^{2}+1-4\chi =0 ,\ \ \ {\left(2\chi -1\right)}^{2}=0,\ \ \ \chi =0.5 ,\ \ \ {\left({n}_{\mu =1}\right)}^{2}=0.5\sqrt{3\left(1-{\nu }^{2}\right)}\cdot \frac{r}{t}$$

Similarly, through Eqs. (4) and (11) we have

15 $${\left({n}_{\mu =0.5, \mu =2}\right)}^{2}=0.32\sqrt{3\left(1-{\nu }^{2}\right)}\cdot \frac{r}{t}$$

Equations (14) and (15) mean that since on whole perimeter the circumferential periodic buckling wavenumber *n* should be an integer, for each *n*, the nonaxisymmetric periodic ECBS value of the circumferential closed-loop eigenmode is the same as that of the axisymmetric ECBS of the longitudinal open-loop eigenmode only at one proper corresponding point, i.e. *σ*^*sc*^_*xcri *_*− σ*^*so*^_*xcri*_ = 0, as follows:

16 $$\frac{{\left({\sigma }_{xcri}^{sc}\right)}_{\mu =1}}{E}=\frac{{\sigma }_{xcri}^{so}}{E}=\frac{1}{2\cdot {n}^{2}} ,\ \ \ \frac{{\left({\sigma }_{xcri}^{sc}\right)}_{\mu =0.5, \mu =2}}{E}=\frac{{\sigma }_{xcri}^{so}}{E}=\frac{1}{3.125\cdot {n}^{2}}$$

**Figure 1:**
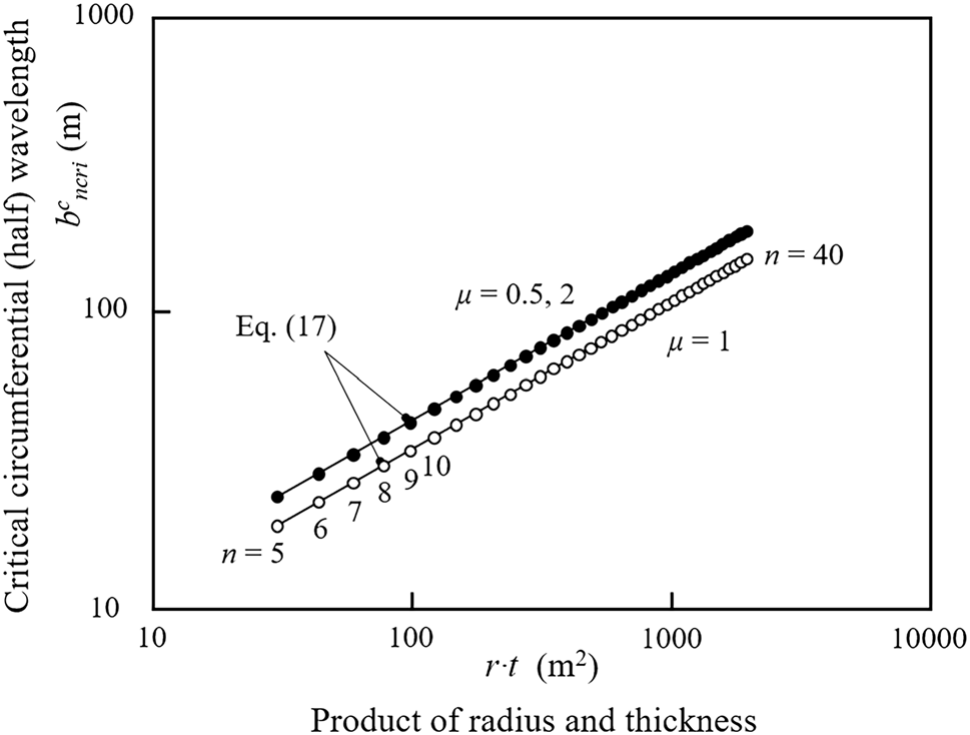
Double-logarithmic plot of the power-law relationship between the critical circumferential wavelength and the product of the radius and thickness of the nonaxisymmetric periodic elastic buckling of a thin-walled perfectly circular cylindrical shell under uniform longitudinal compression. The white and black dots represent the three wave aspect ratios and indicate the biunique values relative to different circumferential periodic buckling integer wavenumbers, respectively.

Otherwise, *σ*^*sc*^_*xcri *_*− σ*^*so*^_*xcri*_ > 0. Consequently, as shown in Fig. 1, the corresponding critical circumferential half-wavelength *b*^*c*^_*ncri*_ is:

17 $${\left({b}_{ncri}^{c}\right)}^{2}={C}_{0}{C}_{\mu }\cdot \left(r\cdot t\right) ,\ \ \ {C}_{0}=\frac{{\pi }^{2}}{\sqrt{3\left(1-{\nu }^{2}\right)}} ,\ \ \ {C}_{\mu }={2}_{\mu =1},\cdots , {3.125}_{\mu =0.5, \mu =2},\cdots$$

Here, the square of the positive and negative critical circumferential wavelengths can be understood as the corresponding clockwise or counterclockwise center angle, which has no substantive difference to the solution based on the whole perimeter.

### Mathematical uniqueness of the Donnell equation solution

To further comprehend the different applicability of the longitudinal open-loop eigenmode and circumferential closed-loop eigenmode, it is necessary to retrospect the mathematical uniqueness and physical significance of the solution of the Donnell equation. Equations (2), (3), and (5) can be expressed in a more general doubly sinusoidal eigenfunction form as follows:

18 $$w={w}_{0}\mathrm{sin}\left(\alpha x\right)\mathrm{sin}\left(\beta y\right)$$

where *α* = *mπ*/*L*, *β* = 0 for the axisymmetric form; *α* = *mπ*/*L*, *β* = *π*/*b*^*c*^ for the nonaxisymmetric periodic form of the longitudinal open-loop eigenmode; or *α* = *π*/*λ*, *β* = *π*/*b*^*c*^_*n*_ for the nonaxisymmetric periodic form of the circumferential closed-loop eigenmode, or any other pair of parameters *α* and *β*. Similarly, by substituting Eq. (18) into Eq. (1), the following equation is obtained:

19 $${w}_{0}\left[D{\left({\alpha }^{2}+{\beta }^{2}\right)}^{4}+\frac{Et}{{r}^{2}}{\alpha }^{4}-t\cdot {\sigma }_{x}^{s}\cdot {\left({\alpha }^{2}+{\beta }^{2}\right)}^{2}\cdot {\alpha }^{2}\right]\mathrm{sin}\left(\alpha x\right)\mathrm{sin}\left(\beta y\right)=0$$

Similar to Eq. (6), the expressions within the brackets in Eq. (19) must be zero, the general solution of ECBS (*σ*^*s*^_*xcri*_) is:

20 $${\sigma }_{xcri}^{s}=\frac{E{t}^{2}}{12\left(1-{\nu }^{2}\right)}\cdot \frac{{\left({\alpha }^{2}+{\beta }^{2}\right)}^{2}}{{\alpha }^{2}}+\frac{E}{{r}^{2}}\cdot \frac{{\alpha }^{2}}{{\left({\alpha }^{2}+{\beta }^{2}\right)}^{2}}$$

Furthermore, by introducing a parameter *ψ* = (*α*^2^ + *β*^2^)^2^/*α*^2^, Eq. (20) can be rewritten as

21 $${\sigma }_{xcri}^{s}=\frac{E{t}^{2}}{12\left(1-{\nu }^{2}\right)}\cdot \psi +\frac{E}{{r}^{2}}\cdot \frac{1}{\psi }$$

Taking the derivative d*σ*^*s*^_*xcri*_/d*ψ* as zero, the minimization can be expressed as follows:

22 $$\frac{\mathrm{d}\left({\sigma }_{xcri}^{s}\right)}{\mathrm{d}\psi }=\frac{E{t}^{2}}{12\left(1-{\nu }^{2}\right)}-\frac{E}{{r}^{2}}\cdot \frac{1}{{\psi }^{2}}=0 ,\ \ \ \psi =\frac{\sqrt{12\left(1-{\nu }^{2}\right)}}{rt}$$

Therefore, the general solution of the ECBS (*σ*^*s*^_*xcri*_) of the Donnell equation is:

23 $${\sigma }_{xcri}^{s}=\frac{E}{\sqrt{3\left(1-{\nu }^{2}\right)}}\cdot \frac{t}{r}$$

The derivation above indicates that the Donnell equation has a general solution if *α* and *β* satisfy the following conditions:

24 $$\frac{{\left({\alpha }^{2}+{\beta }^{2}\right)}^{2}}{{\alpha }^{2}}=\frac{\sqrt{12\left(1-{\nu }^{2}\right)}}{rt}$$

Therefore, for the axisymmetric form of *α* = *mπ*/*L* and *β* = 0, although it is a longitudinal open-loop eigenmode, only a biunique relationship exists in Eq. (24) as follows:

25 $${\alpha }^{2}={\left(\frac{m\pi }{L}\right)}^{2}=\frac{\sqrt{12\left(1-{\nu }^{2}\right)}}{rt} ,\ \ \ L=m\pi \cdot \sqrt{\frac{rt}{\sqrt{12\left(1-{\nu }^{2}\right)}}}$$

However, for the current elastic nonaxisymmetric periodic form (*α* = *mπ*/*L* and *β* = *π*/*b*^*c*^) of the longitudinal open-loop eigenmode, Eqs. (4) and (23) are uniquely determined by the relationship in Eq. (24) and implies that relative to a single *α* or *β*, the solution is not unique. Nevertheless, by designing a circumferential closed-loop eigenmode with the concept of a whole perimeter complete integer periodic waveform that can precisely satisfy the elastic buckling physical features of the ideal circumferential deformation of a thin-walled perfectly circular cylindrical shell under uniform longitudinal compression, that is, *α* = *π*/*λ* and *β* = *π*/*b*^*c*^_*n*_, the solution of the Donnell equation can then be uniquely determined by the relationships in Eqs. (16) and (17), respectively.

### Physical authenticity of the competing eigenmode of the Koiter circle

Furthermore, using Eqs. (21) and (23), we obtain:

26 $$\frac{E}{\sqrt{3\left(1-{\nu }^{2}\right)}}\cdot \frac{t}{r} =\frac{E{t}^{2}}{12\left(1-{\nu }^{2}\right)}\cdot \psi +\frac{E}{{r}^{2}}\cdot \frac{1}{\psi }$$

By introducing the parameter *C* = *t‧r* / √ [3(1 − *ν*^2^)] and inspecting Eq. (26), as follows:

27 $${\left(\frac{1}{2}\cdot C\psi \right)}^{2}-C\psi +1=0 ,\ \ \ {\left(\frac{1}{2}\cdot C\psi -1\right)}^{2}=0 ,\ \ \ \frac{1}{2}\cdot C\psi =1$$

Therefore, if the buckled form is not axisymmetric, then:

28 $${\alpha }^{2}+{\beta }^{2}=\sqrt{\frac{2}{C}} \cdot \alpha$$

Equation (28) is identical to Eq. (3.17.59) in Koiter’s book^21^ and Eq. (14.35) in Calladine’s book^22^ and is the general equation for the contour of *C*(*α*, *β*) in the *α*-*β* plane. The contour is known as the generalized Koiter circle, which means that one *β* value corresponds to two different *α* values.

Here, more generally using the concept of the aspect ratio of the buckling wave pattern, *μ* = *α*/*β*, Eq. (28) becomes:

29 $$\mu +\frac{1}{\mu }=\sqrt{\frac{2}{C}} \cdot \frac{1}{\beta }$$

As can be observed, the left-hand side of Eq. (29) reaches a minimum of 2 when *μ* = 1, and the value increases symmetrically when *μ* is greater than or less than 1. This also implies that, except for *μ* = 1, the same value on the left-hand side corresponds to two different values of the wave aspect ratio (*μ* > 1 vs. *μ* < 1), which can only appear one at a time, not at the same time, physically. Consequently, it can be further inferred that, corresponding to the initiation of elastic nonaxisymmetric periodic buckling of thin-walled perfectly circular cylindrical shells under uniform longitudinal compression, there is no so-called competing eigenmode embodied in the physical relation characterized by the Koiter circle.

## Results and discussion

Although it is still based on the classical static, linear, small-deflection theory of elastic critical buckling of thin-walled perfectly circular cylindrical shells under uniform longitudinal compression, the nonaxisymmetric periodic buckling solution of the circumferential closed-loop eigenmode uniquely defines the critical circumferential wavelength or wavenumber and wave aspect ratio that initially appear on the entire perimeter. In a broader sense, to grasp the essence of the physical phenomenon of elastic buckling, by rationally establishing the explicit geometric concept of the critical circumferential wavelength or wavenumber, the principal differences in the elastic critical buckling models of longitudinal compressed thin-walled structures can be clearly distinguished. Based on this perspective, it is understood that for a longitudinal compressed slender column model, there is no transverse wavelength or wavenumber that can be found; that is, they are all zero; and that for a uniform longitudinal compressed rectangular thin flat plate model, there is only a half transverse wave always adaptively occurring on the whole plate width, that is, its critical transverse half wavelength is always the plate width; and that for a uniform longitudinal compressed thin-walled perfectly circular cylindrical shell model, its axisymmetric buckling form has no circumferential wave, whereas its nonaxisymmetric periodic buckling form has the critical circumferential half wavelength determined by Eq. (17), and the corresponding circumferential periodic wavenumber determined using Eqs. (14) or (15).

In summary, even for the elastic buckling of uniform longitudinal compressed thin-walled perfectly circular cylindrical shells, the linear-elastic mathematical solution originating from Euler’s analytic approach can serve as an effective reference frame for workable engineering solutions.

### Comparison of ECBS between axisymmetric buckling and nonaxisymmetric periodic buckling

**Figure 2:**
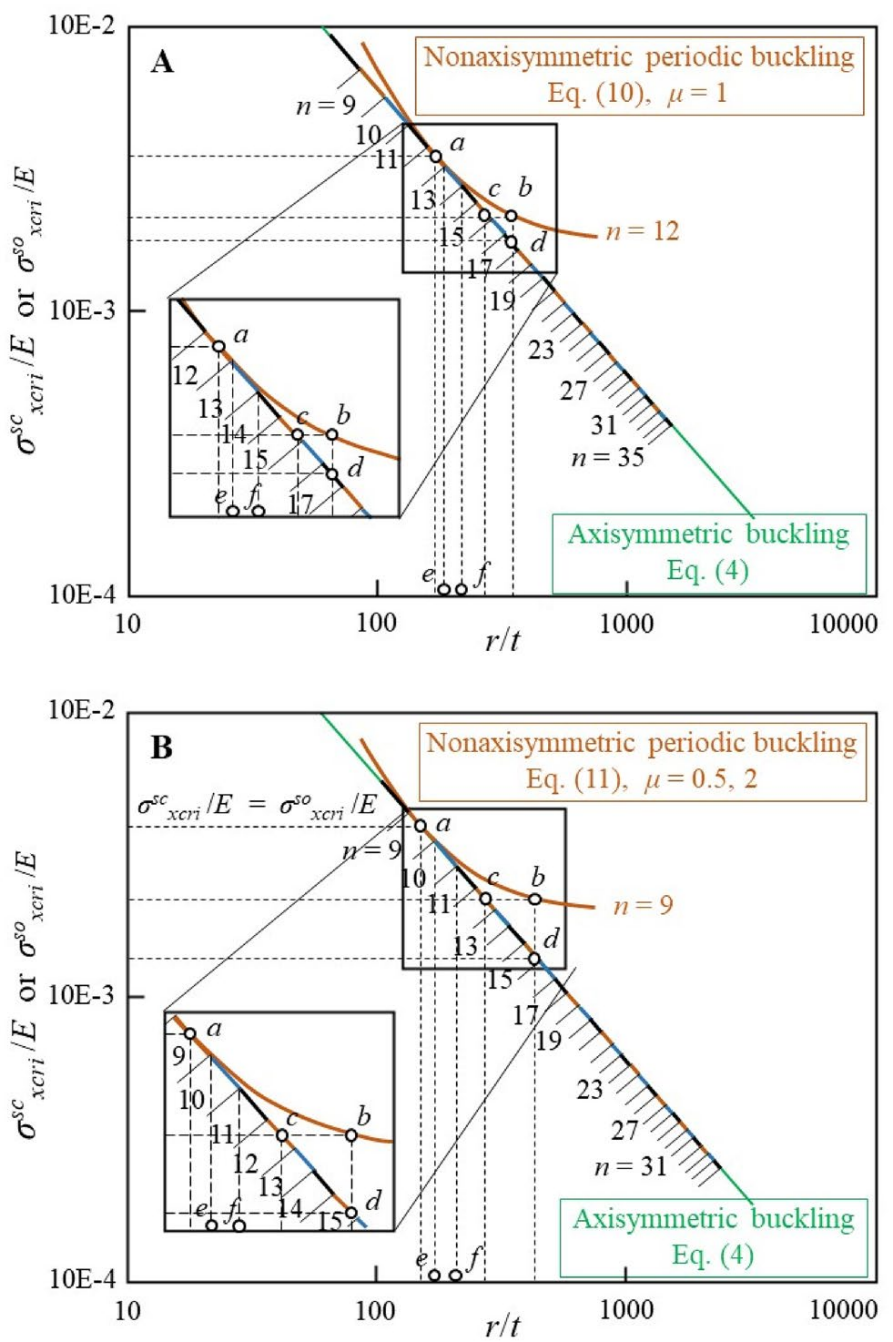
Double-logarithmic plot of the theoretical relationship between ECBS normalized with respect to *E* (*σ*^*sc*^_*xcri*_/*E* or *σ*^*so*^_*xcri*_/*E*) and the radius/thickness ratio (*r*/*t*) of thin-walled perfectly circular cylindrical shells under uniform longitudinal compression. In both (**A**,**B**), (I) the green straight line represents the axisymmetric ECBS calculated using Eq. (4); (II) the black, blue, and brown segments represent the nonaxisymmetric periodic ECBS of the circumferential closed-loop eigenmode calculated using Eqs. (10) or (11), which are the closest values to axisymmetric buckling; (III) dot *a* represents the ECBS of *n* = 12 (or *n* = 9) of the elastic nonaxisymmetric periodic buckling, which is the same as that of axisymmetric buckling and is calculated using Eq. (16); (IV) dot *b* represents the ECBS of *n* = 12 (or *n* = 9) of the elastic nonaxisymmetric periodic buckling, which is quite different from that of axisymmetric buckling and calculated using Eqs. (10) or (11); (V) dot *c* has the same ECBS value as dot *b* (*n* = 12 or 9), is closest to the axisymmetric ECBS, and is obtained by *n* = 15 (or *n* = 12); (VI) dot *d* has the same radius/thickness ratio value as dot *b* (*n* = 12 or 9), is closest to the axisymmetric ECBS, and is obtained by *n* = 17 (or *n* = 15); thus, it can be understood theoretically that from dot *b* to dot *c* or *d*, the nonaxisymmetric periodic ECBS must correspond to different circumferential wavenumber *n* values with a biunique relationship; (VII) the variation range of the radius/thickness ratio between dots *e* and *f* represents the corresponding nonaxisymmetric periodic ECBS range of only one circumferential wavenumber, *n* = 13 (or *n* = 10); and (VIII) by comparing (**A**) and (**B**) (also shown in Fig. 1) shows that, for the same circumferential wavenumber *n*, the necessary critical circumferential wavelength is the shortest when its wave aspect ratio *μ* is 1.

It can be seen from Eq. (4) and Fig. 2, the axisymmetric ECBS is a monotonic continuous function of the radius/thickness ratio, which is most likely to occur earliest because it is not physically constrained by circumferential wave deformation, in contrast to nonaxisymmetric periodic buckling. Nevertheless, for the elastic nonaxisymmetric periodic buckling of the circumferential closed-loop eigenmode and for each of the circumferential periodic buckling wavenumbers *n*, the ECBS is identical to the axisymmetric ECBS at only one proper corresponding point (as dot *a* shown in Fig. 2) and closest to the axisymmetric ECBS within a very small corresponding range (as the *r*/*t* range between dots *e* and *f* shown in Fig. 2). In other words, the part whose ECBS is closest to the axisymmetric ECBS is most likely to buckle; however, for different radius/thickness ratios with relatively large intervals, theoretically, their integer circumferential periodic buckling wavenumber *n* should be different to accommodate the biunique requirement of the most easily deformable continuous condition of the circumferential wave on the whole perimeter, such as dots *a* to *c* or *d*, as shown in Fig. 2. In addition, a comparison of Fig. 2**A** and **B** (also shown in Fig. 1) indicates that for the same circumferential periodic buckling wavenumber *n*, the necessary critical circumferential wavelength is the shortest when its wave aspect ratio is equal to 1 (*μ* = 1.0).

Consequently, for the elastic buckling solution of thin-walled perfectly circular cylindrical shells under uniform longitudinal compression studied herein, the fundamental understanding of its axisymmetric buckling is not different from the previous conventional solution; that is, all quantities continuously change according to the radius/thickness ratio. However, the fundamental understanding of its nonaxisymmetric periodic buckling differs substantially; that is, the geometric concepts of the critical circumferential wavelength or integer wavenumber on the entire perimeter and the wave aspect ratio are newly defined and utilized. Obviously, the elastic eigenmodes of the nonaxisymmetric periodic buckling with different *n* values are sequentially ordered according to their radius/thickness ratio (Fig. 2), rather than the disordered multiple-repeated-competing eigenmodes, as previously understood, as well as qualitatively described by Koiter, that they can interact in a nonlinear fashion, both to reduce the load-carrying capacity and to provide highly unstable postbuckling behavior^14,21^. This different understanding may evolve an approach that can truly interpret the discrepancy between theoretical predictions and experimental measurements and will be discussed next.

### An attempt to interpret discrepancy between theoretical and experimental buckling loads

As we known that all current preliminary design methods of longitudinal compressed thin-walled cylindrical shells including NASA Space Vehicle Design Criteria (SP-8007)^19^ and ECSS Buckling Structures Handbook^20^ are based on the so-called ‘lower bound design philosophy’, by which they recommend the use of a KDF, which corresponds to the worst type of initial geometric imperfections, to multiply the classical buckling load to obtain a lower bound to all available experimental data to provide a safe buckling load prediction for the cases where the total weight and cost of the structure is of no concern. However, in cases where the design is weight-critical, one is usually forced to accept a smaller margin of safety; hence, a more refined method of structural optimization design and analysis is requited^23^. The same situation can also be extended to the currently available large-scale and complex engineering numerical simulation design by using ‘perfect structure’ and KDF, although the magnitude of KDF is largely unknown, mainly due to initial geometric imperfections.

It is worth mentioning that in the early 1960s, an experimental measurement study supported by NASA confirmed the following features^24^: (a) with proper care in manufacturing and testing, values of the buckling stresses can be obtained, which are much higher than those usually found; (b) for the displacement forms tested, small departures from the initial straightness lower the buckling stress and that the effect for inward displacements is greater than that for outward displacements; (c) if the outward displacements are increased, the value of the buckling stress again increases until it reaches essentially the same value as that of the initially straight cylinder; and (d) the constant curvature and sine wave shapes give essentially the same values of buckling stress for larger values of initial displacement.

On the other hand, in some recent studies^8,25^, some skepticism was raised for assuming initial geometric imperfections as stress-free periodic geometric perturbations which have essentially classical buckling mode waveforms and play a striking role in reducing the shell failure load below classical prediction, and could be imparted to the shell as initial eigenmode forms during fabrication, that are open to question. In other words, it is reasonable to postulate a crooked but initially stress-free column; however, it is unreasonable to postulate a plate or shell, because in both cases, it is not possible to impose a double periodic deformation pattern on an initially flat or cylindrical sheet without incurring membrane stresses due to a change in the Gaussian curvature, or whether such hypothetical imperfections are representative of the observable initial geometric imperfections in real shells.

The initial geometric imperfections were considered to play a significant role in degrading the elastic buckling load. However, thus far, it is unclear what type and how it affects ECBS. Properly discriminating and identifying the types and real functions of initial geometric imperfections will help to survey the following significant issues: (a) rationally interpret the discrepancy between theoretical and experimental buckling loads, (b) provide a reasonable and accurate analysis method for sorting out the experimental wide scatter results, and (c) provide appropriate modeling specifications for the initial geometric imperfections of the modern large-scale and complex numerical simulation model in engineering practice.

**Figure 3:**
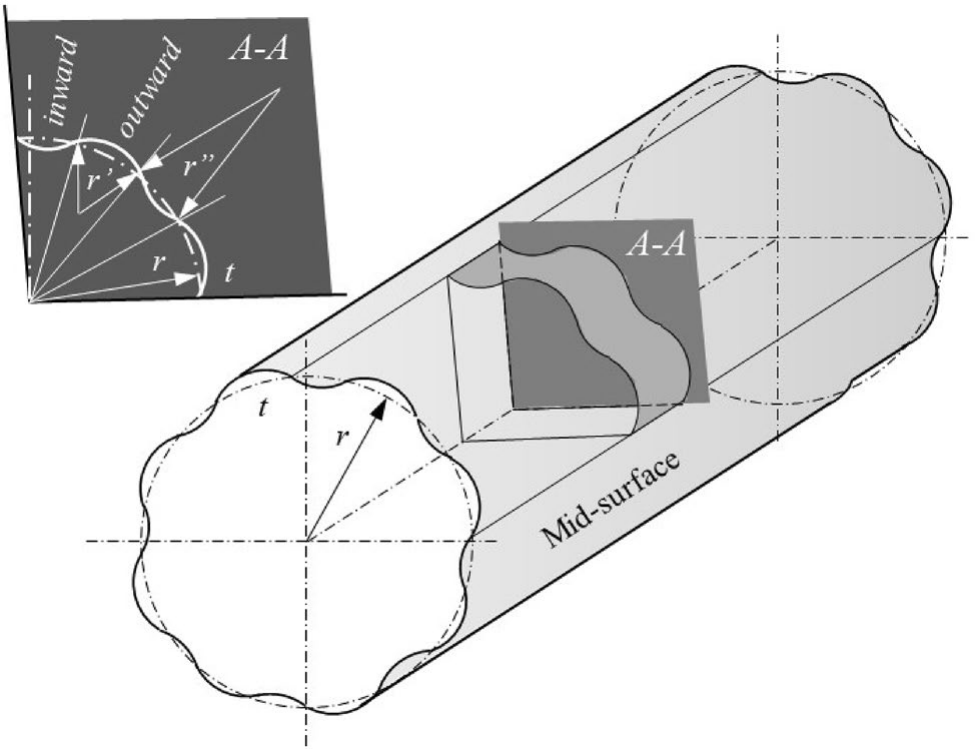
The schematic of the mid-surface distribution of the initial geometric imperfection in an actual isotropic circular cylindrical shell was simplified according to the realistic precisely measured information collected in the Delft databank^26–31^. (I) *r* and *t* are the nominal radius and thickness, respectively. (II) The geomorphic characteristics of a typical initial geometric imperfection can be simplified to longitudinally generated circumferential local outward displacements (longitudinal continuous mountain ranges) and inward displacements (longitudinal continuous river valleys). (III) The curvature (1/*r*’) of the circumferential local outward displacement corresponds to an increase compared to the curvature of its nominal arc (1/*r* < 1/*r*’). (IV) The curvature (1/*r*”) of the circumferential local inward displacement corresponds to a decrease compared with the curvature of its nominal arc (1/*r* > 1/*r*”).

After decades of painstaking investigation and collection work, the research team in Delft established an open databank (Initial Imperfection Data Bank) with realistic information to present the characteristics of the precisely measured surface distributions of initial geometric imperfections in detail, related to the different sizes and fabrication processes of the actual full-scale isotropic circular cylindrical shell with or without integral ring- and stringer-stiffeners^26–31^. From the three-dimensional plots of the actual measured surface distributions of the initial geometric imperfections, it can be seen that most of them exhibit geomorphic characteristics similar to the longitudinally generated continuous mountain ranges and river valleys. A schematic of the mid-surface distribution of the initial geometric imperfection in an actual isotropic circular cylindrical shell simplified according to the realistic precisely measured information collected in the Delft databank^26–31^ is shown in Fig. 3.

From Fig. 2 and Eqs. (7)–(16), it can be seen that for the thin-walled circular cylindrical shell under uniform longitudinal compression, regardless of the elastic deformation influence during loading, the local initial circumferential outward displacement corresponding to an increase in curvature compared to that of its nominal arc, as shown in Fig. 3, locally increases the nonaxisymmetric periodic ECBS of that portion relative to the nominal one. In contrast, in the same situation, the local initial circumferential inward displacement corresponding to a decrease in curvature compared to that of its nominal arc locally decreases the nonaxisymmetric periodic ECBS of that portion relative to that of the nominal one. Simultaneously, the requirement of the local critical circumferential wavelength should be satisfied.

**Figure 4:**
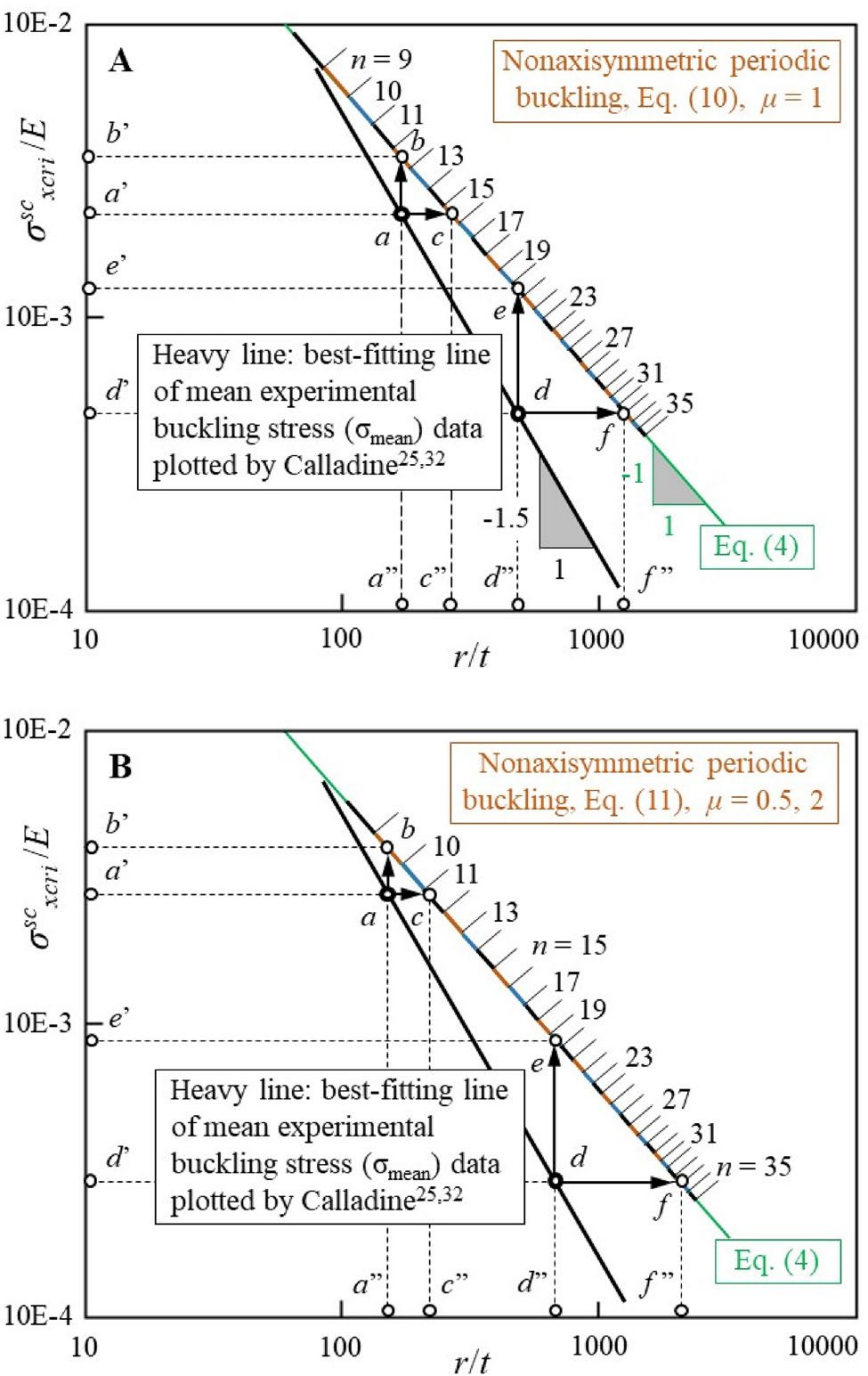
Double-logarithmic plot comparing the experimental and theoretical relationships between the ECBS normalized with respect to *E* (*σ*^*sc*^_*xcri*_/*E*) and the radius/thickness ratio (*r*/*t*) of thin-walled circular cylindrical shells under uniform longitudinal compression. (I) The heavy straight line has a slope of − 1.5 is the best-fitting line of the mean experimental buckling stress data plotted by Calladine^25,32^. (II) For convenience, the thickness *t* is considered constant (unit length of radius *r*). (III) Dot *a* (or *d*) on the heavy straight line is imagined experimental data corresponding to the nominal *r*/*t* indicated by dot *a*’’ (or *d*’’). (IV) The predicted ECBS of the nominal *r*/*t* of dot *a*’’ (or *d*’’) is indicated by dot *b*’ (or *e*’), which corresponds to dot *b* (or *e*) calculated using Eqs. (4) and (10) or (11), respectively. (V) The experimental measurement ECBS of the nominal *r*/*t* of dot *a*’’ (or *d*’’) is indicated by dot *a*’ (or *d*’), which corresponds to dot *c* (or *f*) and is calculated using Eqs. (10) or (11), where it should be derived from the local actual *r*/*t* indicated by dot *c*’’ (or *f*’’) for a nominally identical circular cylindrical shell, which may be produced because of the initial geometric imperfections of its circumferential local inward displacement or because of the similar geometric imperfections that appear and amplify during the loading process. (VI) Owing to, in general, the rare occurrence and consideration of unintentional axisymmetric initial geometric imperfections, such as that similar to corrugated pipe, the actual or experimental elastic critical buckling modes of thin-walled circular cylindrical shell under uniform longitudinal compression indicated by dot *c* (or *f*) will basically be the nonaxisymmetric periodic buckling mode that is corresponded to the increase in its local actual *r*/*t* (dot *c*’’ or *f*’’) relative to nominal *r*/*t* (dot *a*’’ or *d*’’) and its proper local critical circumferential wavelength defined by their unique circumferential wavenumber and wave aspect ratios, as shown in (**A**) and (**B**) respectively. (VII) The best-fitting line of the mean experimental buckling stress data with slope − 1.5 implies that as the experimental nominal *r*/*t* increases (dot *a*’’ to *d*’’), the local actual *r*/*t* increment caused by its circumferential local inward displacement will increase by an average of 1.5 power relative to its nominal *r*/*t* increment, as shown by dot *d*’’ to *f*’’ relative to dot *a*’’ to *c*’’. (VIII) From the above viewpoint, it can be further inferred that except for the case of axisymmetric initial geometric imperfections, the axisymmetric buckling mode considered only occurs in the following two situations: elastic axisymmetric buckling in the thin-walled perfectly circular cylindrical shell and plastic axisymmetric buckling, where the material yield stress is much lower than the predicted and actual or experimental ECBSs.

Based on the above inferences, an attempt was made to physically interpret the discrepancy between the theoretical and experimental buckling loads of thin-walled circular cylindrical shells under uniform longitudinal compression, through the comparisons shown in Fig. 4. The interpretation is that: (a) it is reasonable to believe that the initial geometric imperfections of actual circular cylindrical shell are inevitably composed of their circumferential local outward and inward displacements generated longitudinally; (b) in where the circumferential local inward displacement corresponds to a decrease in curvature compared to that of its nominal arc; (c) the decrement of local curvature will locally decrease the nonaxisymmetric periodic ECBS of that portion relative to nominal one; (d) the locally decreased nonaxisymmetric periodic ECBS will trigger its corresponding nonaxisymmetric periodic buckling mode with proper local critical circumferential wavelength defined by their unique circumferential wavenumber and wave aspect ratios; (e) thus, it is not difficult to infer that the wide scatter of elastic buckling load in the experimental results is mainly due to the scatter of the local curvature variation caused by the circumferential local inward displacement; (f) and from the average of the actual experimental results^25,32^, it can be inferred that the local actual *r*/*t* variation caused by its circumferential local inward displacement will decrease by an average of 1.5 power relative to that of its nominal *r*/*t*.

The interpretation of the degradation of the experimental elastic buckling load presented herein also enables us to rationally deal with seemingly disorganized experimental results. In addition to the nominal radius/thickness ratio, theoretical prediction, and experimentally obtainable elastic buckling loads, the following observation and detection of the actual buckling phenomena of thin-walled circular cylindrical shells under uniform longitudinal compression in the period around the buckling occurrence must also be conducted. That is, (a) the axisymmetric buckling mode or the nonaxisymmetric periodic buckling mode, (b) the local critical circumferential wavelength or circumferential wavenumber, and (c) the wave aspect ratios. For the axisymmetric buckling mode, there are no more than the following two situations: one is for elastic axisymmetric buckling in thin-walled perfectly circular cylindrical shells or that with axisymmetric initial geometric imperfections; the other is for plastic axisymmetric buckling, where the material yield stress is much lower than the predicted and actual or experimental ECBSs. Otherwise, for the nonaxisymmetric periodic buckling mode, the corresponding local actual radius/thickness ratio of the geometric imperfections due to circumferential local inward displacement will be speculated by the above-known factors measured in the period around buckling occurrence and the biunique relationship presented herein.

### Complement discussion

Equation (8) is identical to Eq. (3) or Eq. (1) in Hilburger’s reports^33,34^, which was derived for a uniform longitudinal compressed infinitely long simply supported thin curved plate^17^ and used to estimate the static buckling of the thin skin between stiffeners (local skin-pocket buckling) of an aluminum orthogrid stiffener pattern configuration for NASA aerospace vehicle structures. An example of the variation in the ECBS with the corresponding central angle of the circumferential half wavelength calculated using this equation is shown in Fig. 4A of Ji’s study^35^, which implies that the critical circumferential wavelength of elastic buckling is inevitably affected by the circumferential curvature, which should have been comprehended decades ago.

## Conclusion

A detailed but simple linear-elastic mathematical solution for the elastic critical buckling of thin-walled perfectly circular cylindrical shells under uniform longitudinal compression is proposed, which is still based on the traditional longitudinal open-loop eigenmode solution originating from Euler’s approach, but additional consideration of the circumferential closed-loop eigenmode solution of nonaxisymmetric periodic buckling to satisfy the continuous circumferential buckling deformation constrained by a complete integer periodic waveform on its whole perimeter. By providing the sequentially ordered eigenmode solutions of nonaxisymmetric periodic buckling, the precise and biunique geometric conditions of each distinct critical buckling mode can be identified, which enables the long-standing issue of the discrepancy between theoretical and experimental buckling loads to be reexamined from the perspective of the elastic buckling triggered by the longitudinally generated circumferential local inward displacement in geometric imperfections. For the design cases of longitudinal compressed thin-walled cylindrical shells that are more critical for weight control, further precise consideration of the lower bound of the critical buckling load variation in the elastic state may serve as a useful design clue.

## Data Availability

All data are available in the main text.
